# Cytoplasmic retention of dengue virus capsid protein by metformin impairing nuclear transport

**DOI:** 10.1099/jgv.0.002089

**Published:** 2025-03-20

**Authors:** Ian Carlos Puello-Nakayama, Jonathan Hernandez-Castillo, Juan Manuel Castillo, Daniel Talamás-Lara, Selvin Noé Palacios-Rápalo, Rosa María del Ángel

**Affiliations:** 1 Department of Infectomics and Molecular Pathogenesis, Center for Research and Advanced Studies (CINVESTAV-IPN), Mexico City 07360, Mexico; 2 Unidad de Microscopía Electrónica, Laboratorios Nacionales de Servicios Experimentales (LaNSE), Centro de Investigación y de Estudios Avanzados del Instituto Politécnico Nacional (CINVESTAV-IPN), Av. IPN 2508, Alcaldía Gustavo A. Madero (GAM), Mexico city 07360, Mexico

**Keywords:** capsid protein, dengue virus (DENV), metformin, nuclear passive transport, nuclear transport inhibition

## Abstract

Nuclear transport of proteins larger than 60 kDa occurs via energy-dependent active transport, whereas smaller proteins diffuse into the nucleus through nuclear pore complexes via passive nuclear transport. Although the dengue virus (DENV) replication cycle primarily takes place in the cytoplasm, the capsid protein and non-structural protein 5 (NS5) are imported into the nucleus through a nuclear localization sequence-dependent mechanism. However, given its small molecular weight (14 kDa), the DENV capsid protein may also enter the nucleus via passive diffusion. While some drugs primarily inhibit active nuclear transport, few are known to block passive diffusion. Notably, biguanides have been associated with inhibitory effects on passive nuclear transport. Since biguanides such as metformin (MET) exhibit anti-DENV properties, we investigated the effects of MET on the nuclear transport of DENV proteins. Our results suggest that MET induces changes in the nuclear membrane of Huh-7 cells and reduces capsid nuclear localization without affecting NS5 nuclear import. Furthermore, MET treatment did not alter capsid nuclear import in BHK-21 cells. Additionally, mimicking MET’s effects using a non-hydrolyzable ATP analogue increased capsid cytoplasmic retention and decreased DENV-2 replication. Finally, the inhibition of the classical active nuclear transport pathway did not block capsid nuclear transport, suggesting that DENV-2 capsid enters the nucleus in Huh-7 and Vero cells independently of this pathway.

## Introduction

Dengue is a viral disease prevalent in more than 100 countries in tropical and subtropical areas [1]. There is currently no treatment for dengue infection. However, multiple Food and Drug Administration (FDA)-approved drugs have shown *in vitro* and *in vivo* antiviral effects against dengue virus (DENV) and other flaviviruses [2,4]. The DENV replicative cycle generally occurs in the cytoplasm [5]; however, viral proteins such as the RNA-dependent RNA polymerase NS5 [6], the protease NS3 [7] and the capsid protein [8] have been reported in the nucleus during infection.

The nuclear pore complex (NPC) regulates the nuclear transport of cellular and viral proteins [9]. Active and passive pathways can carry out nuclear transport through the NPC [10]. In active nuclear transport, molecules larger than 60 kDa require a nuclear localization sequence (NLS), which are recognized by karyopherins and are transported into the nucleus in a Guanosine Triphosphate (GTP)-dependent process [11]. On the other hand, in passive nuclear transport, molecules smaller than 60 kDa can diffuse through the NPC in a GTP-independent process [12].

The 102 kDa NS5 protein and the 14 kDa capsid protein have been reported to be imported into the nucleus by active nuclear transport during DENV infection [6,13]. Capsid protein is located in the nucleus and nucleolus of infected cells [14]. It has been reported that capsid protein can interact with several nuclear proteins, affecting nucleosome formation and mediating cell apoptosis [15,17]. In this regard, multiple researchers have reported different FDA-approved drugs that inhibit nuclear-cytoplasmic transport, such as the antiparasitic drug ivermectin (IVM) and the lipid-lowering drug atorvastatin (ATV), which block active nuclear transport [2,18]. Nevertheless, little is known about passive nuclear inhibitors.

In this sense, biguanides such as phenformin, a hypoglycaemic drug, have been shown to affect passive nuclear transport, attributed to the fact that biguanides impair mitochondrial energy production, reducing NPC diameter [19]. Moreover, metformin (MET), another biguanide with anti-DENV properties [20,21], promotes changes in the ATP levels [22], resulting in the activation of the AMP-activated protein kinase (AMPK) and reducing the HMG-CoA reductase (HMGCR) activity, affecting the cholesterol pathway synthesis [21], which gives it its antiviral properties, since DENV requires cholesterol during replication [23,24]. However, the effect of MET on the nuclear transport of DENV proteins is unknown. Hence, in this work, we evaluated the effect of MET on the subcellular localization of DENV NS5 and capsid proteins. Considering that MET has an anti-DENV effect, this work is expected to elucidate an additional effect of MET that could contribute to reducing DENV replication.

## Methods

### Cellular culture

The human hepatocarcinoma cell line Huh-7 and Vero cells were cultured in Dulbecco’s Modified Eagle Medium (DMEM) supplemented with glutamine at 2 mM, 7% of FBS, high glucose content (4 g l^−1^), penicillin (50 U ml^−1^) and streptomycin (50 µg ml^−1^) at 37 °C in a humidified atmosphere with 5% CO_2_. The baby hamster kidney-21 cells (BHK-21) were maintained in DMEM supplemented with 8% of FBS, high glucose content (4 g l^−1^), penicillin (50 U ml^−1^) and streptomycin (50 µg ml^−1^) at 37 °C in a humidified atmosphere with 5% of CO_2_.

### Viral infection and plaque assay

The DENV-2 (New Guinea C strain) and DENV-4 (H241) were donated by the Institute of Epidemiological Diagnostic and Reference Dr. Manuel Martínez Báez (InDRE), Mexico. DENV-2 and DENV-4 were propagated using the brains of suckling CD-1 mice (provided by Unidad de Producción y Experimentación de Animales de Laboratorio-UPEAL, CINVESTAV). Extracts from non-infected CD-1 mice brains were used as controls.

The plaque assay protocol reported by Morens D.M. *et al*. was used to determine viral titre [25]. Briefly, BHK-21 cells were cultured at 80% confluence and infected with serial dilutions of DENV-2 in HANK’s balanced salt solution for 2 h at 37 °C with 5% CO_2_. Subsequently, 1 ml of carboxymethylcellulose (CMC) was added and incubated for 5 days at 37 °C with 5% CO_2_. Finally, CMC was removed, and cells were stained with naphthol blue-black to count the plaque-forming units (PFU).

### Drug preparation

A metformin tablet (DABEX) was resuspended in water to obtain a 1.645 Molar stock solution, and aliquots of 100 mM were prepared. AMP-PNP (Sigma #10102547001) was resuspended in water to obtain a 5 mM stock solution. Ivermectin (Sigma #I8898-250MG) was resuspended in DMSO at 2.5 mg ml^−1^ concentration. All stock drugs were stored at −20 °C. Final working concentrations were obtained after serial solutions. The group control cells were treated with water or DMSO as a vehicle.

### Cell viability assay

The toxicity effects of MET or AMP-PNP on the viability of Huh-7 cells were evaluated using the 3-(4,5-dimethylthiazol-2-yl)−2,5-diphenyltetrazolium bromide (MTT) assay (Sigma-Aldrich). In brief, cells were cultured in 96-well flat-bottom plates in complete DMEM and cultured overnight. Huh-7 cells were treated with increasing concentrations of MET (0.1, 0.5, 1, 10, 45 and 90 mM) or AMP-PNP (0.05, 0.1, 0.5, 1 and 5 mM) for 24 h. Then, 5 mg of MTT reagent was weighed and dissolved in 1 ml of 1X PBS. After adding 10 µl of MTT reagent and 100 µl of complete medium to each well, the plates were incubated for 3 h at 37 °C. Formazan crystals were solubilized using DMSO, and the absorbance of each well was measured at 562 and 630 nm to eliminate the background. The OD of the control cells treated with the vehicle was considered 100% viable.

### Nuclei isolation

The Huh-7 cells grown in p100 dishes at 80% were infected with mock or DENV-2 for 24 h and washed three times using 1X PBS. Then, the cells were trypsinized and homogenized in ice-cold hypotonic buffer (20 mM HEPES, 10 mM KCl, 1 mM EDTA, 10% glycerol, 0.5% Triton X-100 and a protease inhibitor cocktail), incubated at 4 °C for 10 min, vortexed for 1 min and centrifuged at 2,000 ***g*** for 1 min. The supernatants corresponding to cytoplasmic fractions were discarded. At the same time, the nuclear pellets were gently resuspended in ice-cold hypotonic buffer and washed three times to remove the remains of the cytoplasmic fraction.

### Scanning electron microscopy

Purified Huh-7 cell nuclei were fixed with 2.5% (v/v) glutaraldehyde in 0.1 M sodium cacodylate buffer pH 7.2 for 1 h at room temperature (25 °C). Then, the nuclei were adhered to coverslips previously coated with Poly-l-lysine solution 0.1% (w/v) in H_2_O for 2 min. After washing, samples were dehydrated with increasing concentrations of ethanol, critically point dried with liquid CO_2_ (31 °C and 1100 psi) using a Samdri 795 apparatus (Tousimis Corp., Rockville, MD) and metalized with gold particles in an ion sputtering device for observation in a JEOL-JSM 6510 LV scanning electron microscope (JEOL Ltd., Tokyo, Japan).

### Immunofluorescence assay

The Huh-7 cells infected with DENV-2 were treated or not with MET 1 mM for 24 h in coverslips placed in 24-well plates. Subsequently, they were fixed with 4% paraformaldehyde for 30 min at 4 °C and permeabilized (0.2% saponin, 1% FBS and 1X PBS) for 30 min at room temperature. The cells were incubated overnight at 4 °C with the antibody of interest (see Table S1, available in the online Supplementary Material). Goat anti-mouse Alexa Fluor 488 and goat anti-rabbit Alexa Fluor 555 (Life Technologies) were used as secondary antibodies, and the nuclei were stained with Hoechst (Santa Cruz). The coverslips were mounted using 3 µl of VECTASHIELD® antifade mounting medium (Vector Laboratories), and their edges were sealed with nail polish. The labelled cells were observed with the Leica TCS SP8 confocal microscope (Leica Microsystems), using control of cells labelled with secondary antibodies to remove the background noise. Images were obtained with the Leica Application Suite X Core Offline v3.3.0 software, and its fluorescence intensity was analysed using the Icy software.

### Cell fractionation

Huh-7 cells were cultured in 6-well plates at 70% or 80% confluence and were infected or not (Mock) with DENV-2 in the presence or not of treatments during 24 h. Cells were infected with DENV at a MOI of 3 in the presence or absence of 1 mM MET treatment for 24 h. Next, cells were detached with trypsin and centrifuged at 300 ***g*** for 5 min, and the pellet was resuspended in 1X PBS to wash the cells. The washing process was done twice. Finally, cells were fractionated following the manufacturer’s instructions for the Abcam fractionation kit (ab109719). The nuclear and cytoplasmic fractions were stored at −80 °C.

### Plasmid transfection

The plasmid containing the NLS of the simian virus 40 (SV40) (KKKRK) with four green fluorescent proteins (GFP) was kindly donated by Dr. Bulmaro Cisneros from the Genetic and Molecular Biology Department of the Investigation and Advanced Studies Centre (CINVESTAV, México). The plasmid was propagated in competent *Escherichia coli* DH5α (Invitrogen), and the purification was done using the Zyppy Plasmid Miniprep Kit (ZYMO Research), following the manufacturer’s instructions. Huh-7 cells were transfected at a 70%–80% confluence by electroporation following the [26,26] protocol with some modifications. Briefly, 1×10^7^ cells were washed with cold 1X PBS and resuspended in 100 µl of OptiMem, and then 5 µg of plasmid DNA was added. The cells and the plasmid mixture were transferred to a 4-mm Gene Pulser Cuvette. The electroporation was done in a Gene Pulser Xcell (BioRad, Germany), with an electric field intensity and pulse length of 170 V and 40 ms in exponential decay. Finally, the cells were cultured in advanced DMEM with 15% FBS, and the transfection was evaluated 48 h later using confocal microscopy.

### Western blot of protein extracts and nuclear fractions

Huh-7 cells were mock-infected or DENV-2-infected at an MOI of 3 in 6-well plates. Then, the cells were washed twice with 1X PBS and treated with Bio-Rad lysis buffer RIPA (25 mM Tris HCl pH 7.6/150 mM NaCl/5 mM EDTA/ 1% Triton X-100/1% sodium deoxycholate/0.1% SDS) and protease inhibitor Roche (cOmplete Protease Inhibitor Cocktail Tablets Cat. No. 11697498001) to extract the total protein. The cellular fraction and total protein extracts were quantified using the bicinchoninic acid (BCA) protein assay (Thermo Scientific). Then, 20–30 µg of protein were separated using SDS-PAGE and were transferred to nitrocellulose membranes (Bio-Rad). Then, membranes were blocked with 10% skim milk in PBST (1X PBS/0.01% Triton X-100) for 1 h at room temperature. The immunoblot was performed with the primary antibody against the protein of interest (see Table S1). For the loading control, the mouse anti-GAPDH antibody was used; for the nuclear fraction control, the mouse anti-laminin A/C antibody was used, and the mouse anti-calreticulin polyclonal antibody was used for the cytoplasmic fraction control. All the primary antibodies were incubated overnight (16–18 h) at 4 °C. As secondary antibodies, goat anti-mouse or anti-rabbit IgG antibodies conjugates with HRP were used with 5% skim milk in PBST and incubated at room temperature for 1 h. Proteins were visualized using the Super Signal West Femto (Thermo Scientific) chemiluminescent substrate (Thermo Scientific), and densitometric analysis was performed with ImageJ software.

### Statistical analysis

Two-way ANOVA with Bonferroni’s multiple comparison test was used to compare the cell viability of treated and untreated cells. The mean fluorescence intensity (MFI) of the pixel values for each region of interest was selected to quantify the fluorescence nucleus–cytoplasmic (Fn/c) ratio. The Fn/c ratio was determined according to Fn/Fc=(Fn-Fb) / (Fc-Fb), where Fb was the background fluorescence. The MFI arbitrary units were expressed as the mean, and the sem was determined in all cases. The Student’s t-test was used to compare the treated and untreated groups in confocal microscopy, densitometric analysis of the bands and plaque assays. In all cases, a *P*≤0.05 was considered statistically significant.

## Results

### Visualization of the nuclear membrane of Huh-7 cells treated with MET by scanning electron microscopy

Previous reports have discussed inhibiting active cellular transport as an antiviral target, given that the nuclear localization of viral proteins plays a crucial role in the replicative cycle, especially in *in vitro* assays using IVM (2.5 µM) or ATV (10 µM) drugs [9,18, 27, 28]. However, little is known about passive transport inhibitors. In this regard, it was reported that the FDA-approved hypoglycaemic drug MET is capable of disrupting passive nuclear transport by reducing the diameter of NPCs by modifying ATP levels [19,29]. Therefore, we decided to evaluate the nuclear membrane surface by scanning electron microscopy (SEM) to identify changes in the Huh-7 cells’ nuclear membrane during MET treatment.

**Fig. 1.**
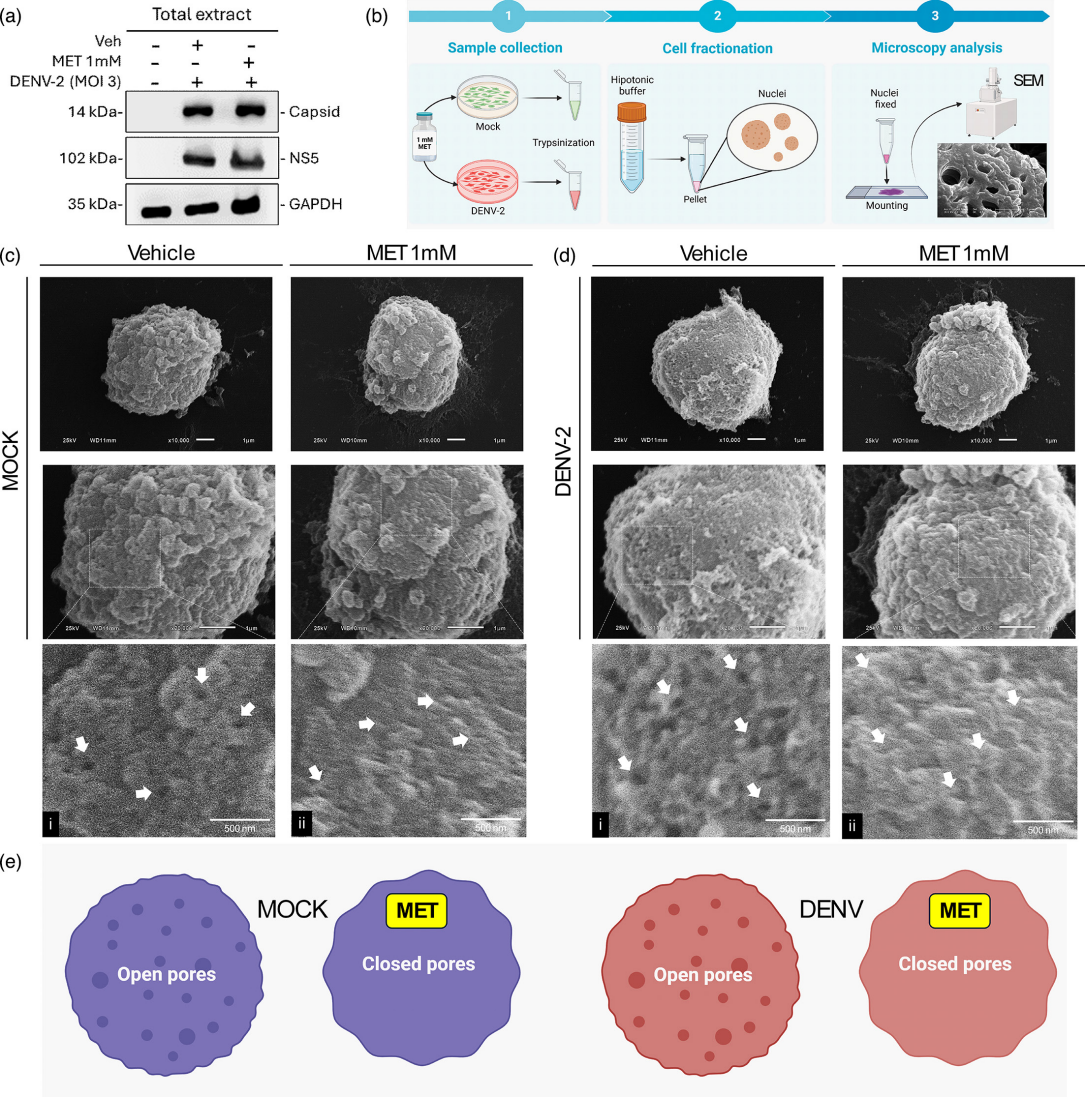
Effect of MET treatment on Huh-7 cell nuclear membrane during DENV-2 infection. (**a**) Western blot analysis of total extract from Huh-7 cells infected with DENV-2 and treated with 1 mM MET for 24 h. Antibodies against capsid and NS5 were used. GAPDH was used as a loading control. (**b**) Workflow for visualization of Huh-7 cell nuclei during MET treatment. (1) Cells infected or not with DENV-2 and treated or not with 1 mM MET were collected. (2) Cell fractionation was performed, and nuclei were isolated in a hypotonic buffer. (3) The pellet corresponding to the nuclei was fixed and prepared for visualization by SEM. (**c**) Magnification of isolated nuclei of mock-infected Huh-7 cells: (i) shows the zoom of the nucleus of mock-infected cells treated with vehicle, and (ii) shows the zoom of the nucleus of mock-infected cells treated with 1 mM MET. (**d**) Magnification of isolated nuclei of DENV-2-infected Huh-7 cells: (i) shows the zoom of the nucleus of DENV-2-infected cells treated with vehicle, and (ii) shows the zoom of the nucleus of DENV-2-infected cells treated with 1 mM MET. White arrows indicate pore-like structures. (**e**) Schematic representation of the effect of MET on NPCs. Scale bar, 1 µm and 500 nm.

First, the cell viability percentage of the MET-treated cells was evaluated. We determined that a 1 mM concentration of MET did not reduce the Huh-7 cell viability (Fig. S1A). We chose the 1 mM MET concentration based on the fact that in previous reports, 1 mM phenformin (another drug of biguanide class) blocks passive nuclear transport [19]. Then, to evaluate the effect of 1 mM MET on the expression of DENV-2 proteins, we performed Western blot assays of total extracts from Huh-7 cells infected with DENV-2 at an MOI of 3 (PFU ml^−1^) at 24 hpi and treated with 1 mM MET for 24 h. Our results showed that 1 mM MET did not affect the amount of NS5 and capsid proteins of DENV-2 (Fig. 1a).

In addition, mock-infected or DENV-2-infected Huh-7 cells were fractionated to recover the nuclei, which were fixed, prepared and observed by SEM; the image of the nuclei at a magnification of ×450 is shown in Fig. S1B. The results in ×10,000 magnification images show pore-like structures in the nucleus of untreated mock-infected cells (Fig. 1c). However, the nucleus of mock-infected cells treated with 1 mM MET appears to have a surface devoid of pore-like structures that exhibit a dark sink, as observed in the untreated mock (Fig. 1c(i) and (ii), indicated by white arrows). Additionally, we observed similar characteristics in the nucleus of untreated DENV-2-infected cells and lower roughness compared to the nuclei of mock-infected cells (Fig. 1d(i)). On the other hand, relevant changes in the nuclei of DENV-2-infected cells treated with 1 mM MET were observed, possessing similar characteristics to untreated and 1 mM MET-treated mock-infected cells (Fig. 1d(ii)), suggesting the possibility that MET impacts nuclear pore aperture, as shown in the schematic Fig. 1e. All these preliminary data suggest that treatment with 1 mM MET could promote physical changes in the nuclear membrane, which could affect nuclear transport during DENV-2 infection.

### Nucleolar localization of DENV-2 capsid is reduced by MET treatment in Huh-7 cells

Given the effect of MET treatment on the nuclear membrane in uninfected and infected cells, we wonder if MET could arrest the nuclear transport of DENV-2 viral proteins. Thus, we analysed the subcellular localization of NS5 and capsid proteins. Briefly, Huh-7 cells were infected with DENV-2 at an MOI of 3 (PFU ml^−1^), and the infection was allowed for 18, 24 and 30 h. Afterwards, the cells were stained with antibodies against viral proteins NS5, capsid and prM. The viral protein prM was used as an infection control because its localization is entirely cytoplasmic.

**Fig. 2.**
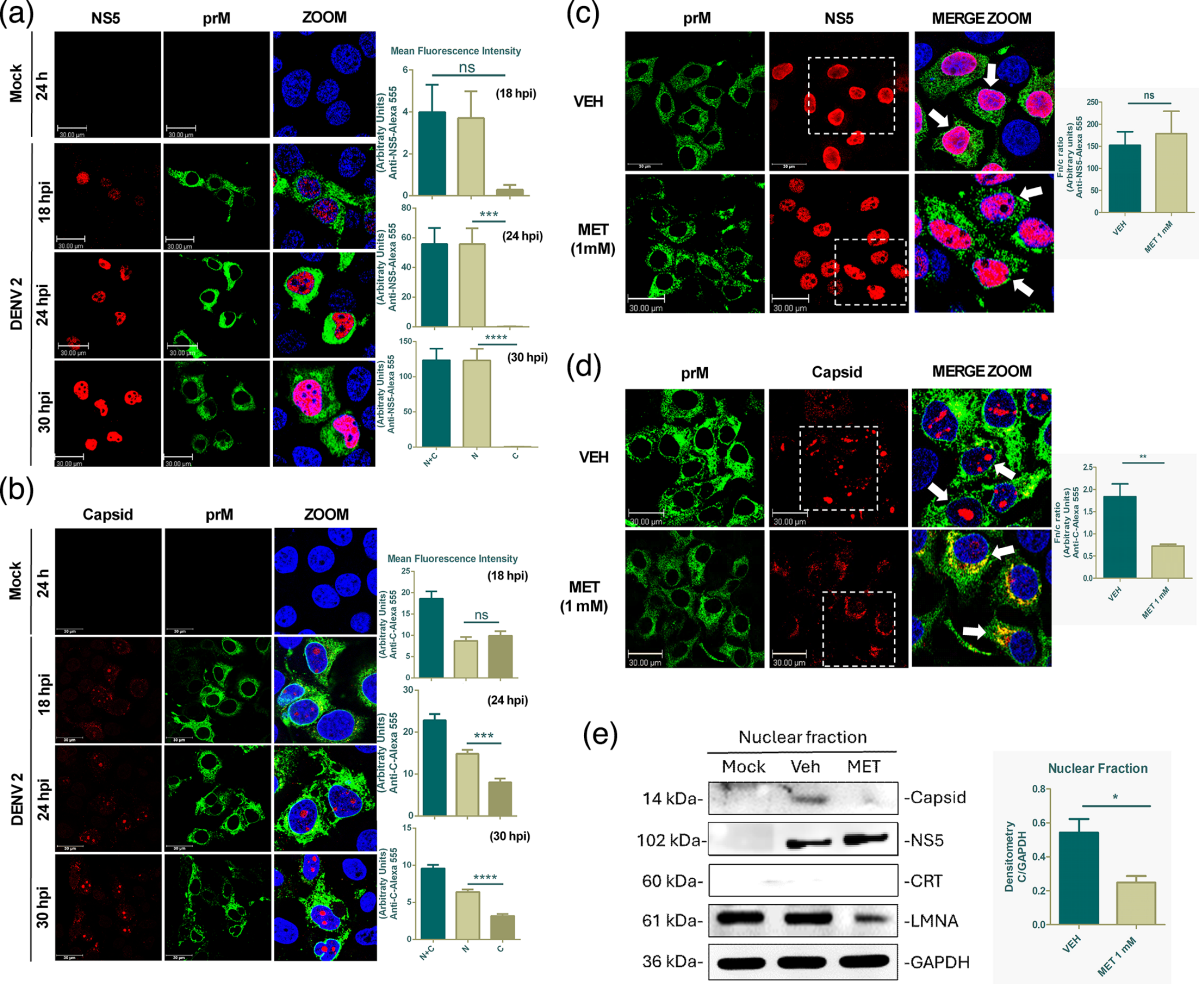
Passive transport inhibition by MET reduces the nuclear import of DENV-2 capsid protein. Infection kinetics of DENV-infected Huh-7 cells to assess viral protein localization at 18, 24 and 30 hpi. NS5 (**a**) and capsid (**b**) were observed in red colour, prM in green colour and cell nuclei in blue colour. The graphs evaluate the MFI of the nuclear, cytoplasmic and nucleus plus cytoplasm fractions. Confocal microscopy of DENV-2-infected Huh-7 cells treated or not with 1 mM MET for 24 h was then performed. NS5 (**c**) and capsid (**d**) localization were evaluated. Fluorescence intensity was evaluated, and the Fn/c ratio was obtained. (**e**) Western blot analysis of the nuclear fraction of Huh-7 cells infected with DENV-2 and treated or not with 1 mM MET for 24 h. CRT and LMNA were used as purity controls, and GAPDH was used as a loading control. Densitometry analysis of the capsid bands was subsequently performed. Data were represented as mean±sem. The Student’s t-test was performed for statistical testing, and *P*<0.05 was considered a significant difference. ns, not significant; *P<0.05; **P<0.01. Scale bar, 30 µm.

Our results showed that NS5 localization is predominantly nuclear at 18 hpi, likewise, at 24 and 30 hpi (Fig. 2a). Nevertheless, the MFI shows a trend in the increase of NS5 in the nucleus (mean 18 hpi=3.7 arbitrary units, 24 hpi=55.7 arbitrary units and 30 hpi=123.1 arbitrary units), suggesting increased replication and translation of viral proteins (Fig. 2a). Similarly, peak levels of capsid protein fluorescence in the nucleus were observed at 18 hpi, increasing its MFI into the nuclei of infected cells (mean, 18 hpi=8.6 arbitrary units, 24 hpi=14.8 arbitrary units and 30 hpi=6.3 arbitrary units) (Fig. 2b). Of note, cytoplasmic capsid fluorescence was decreased at 24 hpi (mean, 8.1 arbitrary units) and 30 hpi (mean, 3.6 arbitrary units) compared to 18 hpi (mean, 9.1 arbitrary units) (Fig. 2b). Based on the infection kinetics results, we concluded that 24 hpi is sufficient to evaluate the effect of pharmacological inhibition on the nuclear transport of NS5 and capsid protein.

In this regard, the results of the nuclear transport inhibition assay with MET results show that NS5 nuclear localization is not impaired after 1 mM MET treatment (Fig. 2c) and the Fn/c ratio indicates no significant reduction between vehicle (152.3±30.49) and MET (178.5±51.06) conditions (Fig. 2c). In addition, the results show that the capsid protein is located mainly in the nucleolus in non-treated cells, as we observed in Fig. 2b. In contrast, during 1 mM MET treatment, the capsid protein was mainly located in the cytoplasmic region, specifically in the perinuclear area (Fig. 2d). The Fn/c ratio analysis revealed a significant reduction of the capsid protein between vehicle (1.836±0.28) and MET (0.7240±0.04) conditions, suggesting that 1 mM MET impairs the nuclear import of the capsid. Additionally, the total MFI of the NS5 and capsid protein was analysed, and we found no significant difference between vehicle and MET, suggesting that the 1 mM MET treatment does not affect the viral protein expression levels, in concordance with the Western blot of total extract shown in Fig. 1a, but promoting the cytoplasmic accumulation of the capsid protein.

Finally, to confirm that MET treatment promotes the nuclear reduction of capsid protein and not NS5, a Western blot analysis was performed after fractionation of DENV-2-infected Huh-7 cells, treated and untreated with 1 mM MET. Lamin A/C (LMNA) and calreticulin (CRT) antibodies were used to control purity for the nuclear and cytoplasmic fraction, and GAPDH was used as a loading control. The results did not show apparent changes in the NS5 bands corresponding to 1 mM MET-treated cell fractions compared to the vehicle (Figs1e  2a). In contrast, we observed a decrease of capsid protein in the nuclear fraction (Fig. 1e) with a concomitant increase in the cytoplasmic fraction during MET treatment compared to the vehicle (Fig. S2A), confirming our indirect immunofluorescence results.

To assess whether the effect of MET on nuclear transport of the capsid protein is consistent across different cell lines, we replicated the MET treatment assay in Vero and BHK-21 cells during DENV-4 infection. In Huh-7 cells infected with DENV-4, we observed an increase in capsid fluorescence in the cytoplasm of infected cells treated with 1 mM MET (Fig. S2B). This was accompanied by a significant reduction in the Fn/c ratio from 1.651±0.15 in the vehicle-treated cells to 0.01894±0.007 in MET-treated cells (Fig. S2C), a pattern similar to what we observed with DENV-2 infection. In the Vero cell line, during DENV-2 infection, MET treatment (1 mM) induced a slight increase in capsid fluorescence in the cytoplasm (Fig. S2D), significantly reducing the Fn/c ratio from 50.24±13.51 in the vehicle-treated cells to 3.782±0.68 in MET-treated cells (Fig. S2E). However, in DENV-4-infected Vero cells, no significant changes were observed between the vehicle-treated (10.25±1.48) and MET-treated (7.916±2.94) groups (Fig. S2D and F). In the BHK-21 cell line, capsid protein localization during both DENV-2 and DENV-4 infection was predominantly nuclear in both treated and control cells (Fig. S3A, B). A slight, non-significant reduction in the Fn/c ratio was observed between the vehicle and MET-treated groups (Fig. S3C, D). Our data suggested a differential effect of MET in the nuclear localization of capsid protein depending on the cellular context but preserved between the two serotypes in the Huh-7 cell line.

### The ATP regenerative system modulation decreases nucleolar localization of DENV-2 capsid protein

Regarding the mechanism of MET, it is well known that the key target is mitochondrial bioenergetic modulation, affecting ATP production through interaction with the respiratory chain complex [22], and cellular ATP modulation leads to alterations in NPC diameter [30]. This mechanism has been linked to the inhibition of passive nuclear transport [19]. Therefore, to test the involvement of ATP in the nuclear import of capsid protein, DENV-2-infected Huh-7 cells were incubated with the non-hydrolysable ATP analogue, AMP-PNP, at a concentration of 0.5 mM, based on our previous cell viability assays (Fig. S3E).

**Fig. 3.**
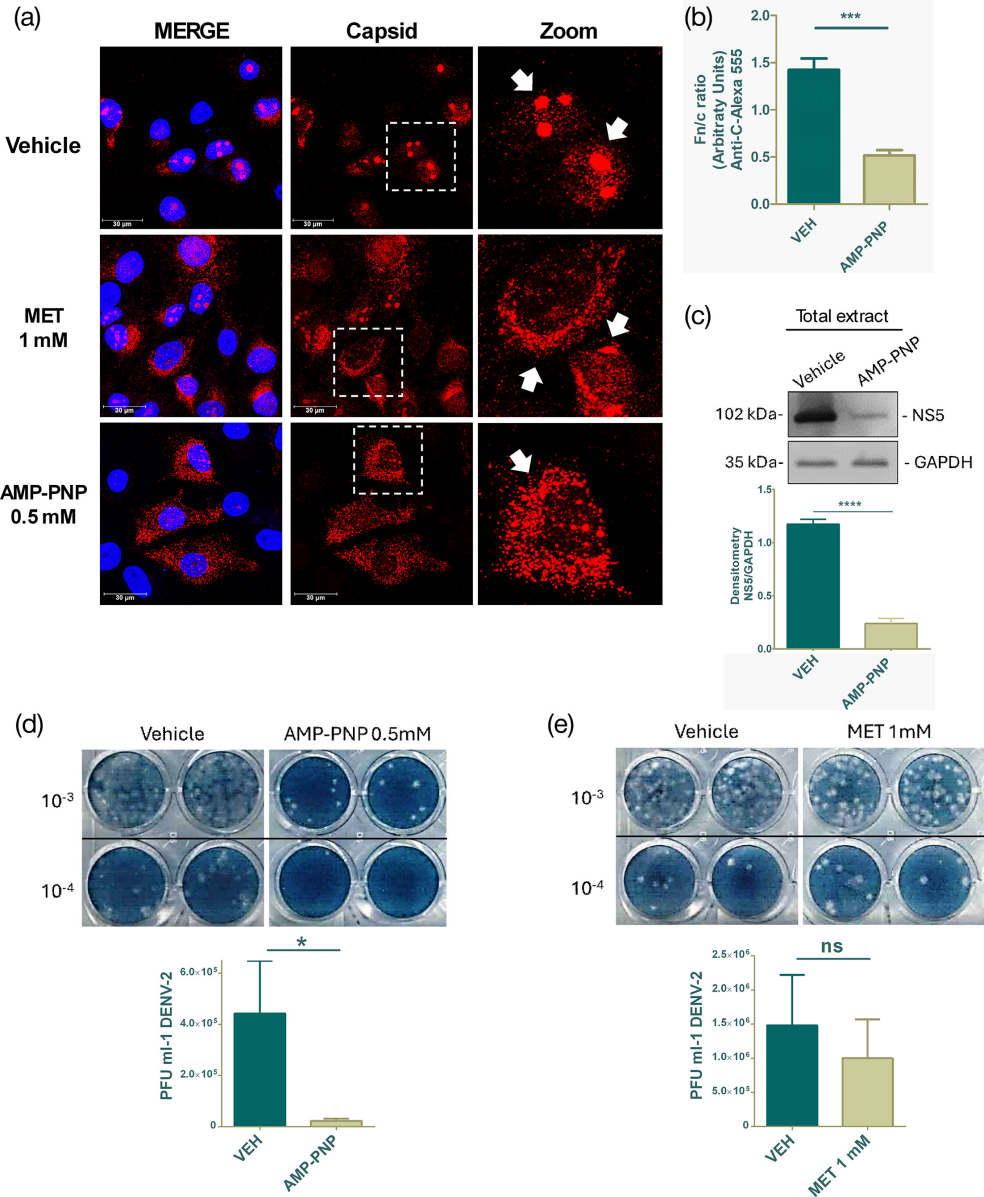
Non-hydrolysable ATP analogue treatment promotes cytoplasmic accumulation of DENV-2 capsid protein. (**a**) Representative confocal microscopy images of DENV-2-infected cells treated with the non-hydrolysable ATP analogue, AMP-PNP. MET-treated cells were used as controls. The white arrows show the cytoplasmic or nuclear localization of the capsid. (**b**) MFI graphic showing the Fn/c ratio between vehicle-treated and 0.5 mM AMP-PNP-treated DENV-2-infected cells. (**c**) Western blot analysis of total extract from Huh-7 cells infected with DENV-2 and treated with 0.5 mM AMP-PNP for 24 h. The graph shows the densitometric analysis of the bands corresponding to the NS5 protein. (**d, e**) Plaque assay of supernatants from DENV-2-infected cells treated with vehicle, AMP-PNP or MET. The graphs represent the viral titre of four independent assays. Data were represented as mean±sem. The Student’s t-test was performed for statistical testing, and *P*<0.05 was considered a significant difference. ns, not significant; *P<0.05; ***P<0.001. Scale bar, 30 µm.

Our results show that treatment with 0.5 mM AMP-PNP reduced capsid accumulation in the nucleolus and nucleus, increasing in cytoplasm similar to MET treatment (Fig. 3a), in comparison with untreated infected cells, resulting in a significant decrease of the Fn/c ratio (Fig. 3b). Regarding the nuclear transport of NS5, AMP-PNP treatment did not disrupt its nuclear import (Fig. S3F). Lastly, we analyse the effect of AMP-PNP on DENV-2 replication. Western blot results showed an evident decrease of viral protein in infected cells treated with 0.5 mM AMP-PNP, compared with vehicle treatment (Fig. 3c). In addition, we measured the viral titre in the supernatants of cells with both treatments. The plaque assay results showed that AMP-PNP significantly reduced the DENV-2 viral titre (Fig. 3d). Conversely, MET treatment at 1 mM concentration did not significantly decrease viral titre (Fig. 3e). Overall, these data suggest an important role for ATP in capsid nuclear transport and DENV-2 replication.

### Active transport inhibition does not affect the nucleolar accumulation of DENV-2 capsid protein

**Fig. 4.**
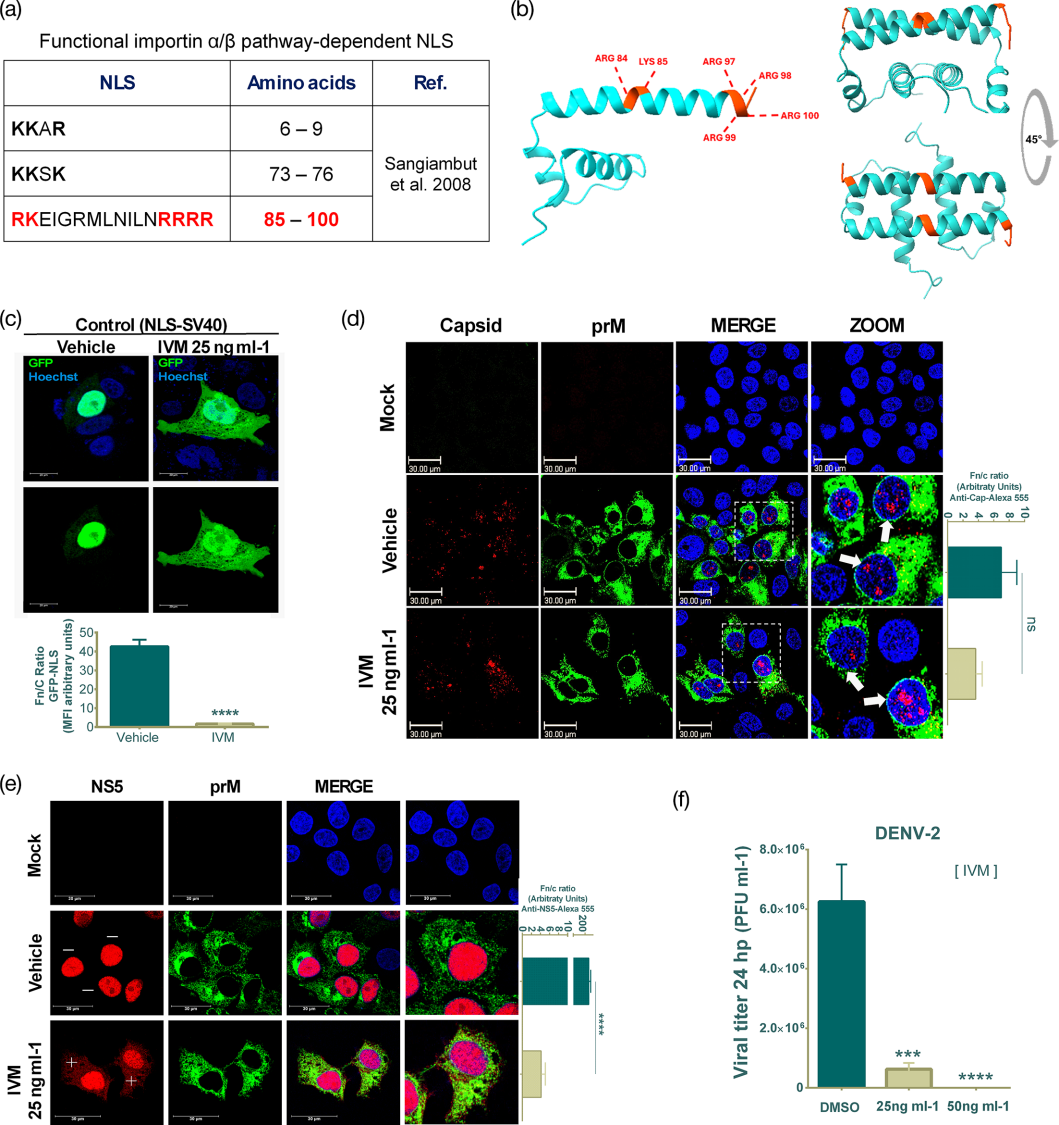
Active transport inhibition does not affect the nuclear import of DENV-2 capsid protein. (**a**) Table of three putative functional NLSs reported in the capsid of DENV. Bipartite NLS is highlighted in red. (**b**) Visualization of bipartite NLS (red colour) in the capsid monomer and homodimer. (**c**) Confocal microscopy images of cells transfected with SV40-x4GFP NLS in the presence of vehicle (DMSO) or 25 ng ml^−1^ IVM, as a control of importin αβ1 pathway-dependent nuclear transport inhibition. (**d**) Confocal microscopy images of DENV-2-infected cells treated with vehicle or IVM. The white arrows show the nuclear localization of the capsid. (**e**) Confocal microscopy images of DENV-2-infected cells treated with vehicle or IVM showing the staining for NS5 protein. (−) indicates no presence, and (+) indicates presence. The graphs evaluate the mean of the Fn/c ratio. Data were represented as mean±sem. The Student’s t-test was performed for statistical testing, and *P*<0.05 was considered a significant difference. (**f**) Plaque assay of supernatants from DENV-2-infected cells treated with vehicle or IVM. The one-way ANOVA was performed for statistical testing, and *P*<0.05 was considered a significant difference. ns, not significant; ***P<0.001, ****P<0.0001. Scale bar, 30 µm.

A previous report using site-directed mutagenesis showed that the capsid protein possesses putative functional NLS specific for the classical nuclear importin *αβ*1 pathway [13] (Fig. 4a). Moreover, to visualize the reported putative NLS exposed in the capsid protein, the dimer and homodimer were modelled via AlphaFold 3.0. In both cases, the putative bipartite NLS (in red colour) was shown to be accessible for nuclear transport receptors (Fig. 4b), supporting the idea that capsid protein is imported by active transport using the importin *αβ*1 pathway [13]. Therefore, we decided to confirm the participation of this pathway in the nuclear transport of capsid protein using the antiparasitic drug IVM, which is a specific inhibitor of active nuclear transport [27]. First, to validate the effect of IVM on active nuclear transport, a plasmid containing the NLS from de simian virus 40 (SV40) (KKKRK) tagged to four GFPs was transfected. The results showed that the GFP signal localization was nuclear in vehicle-treated cells (Fig. 4c). In contrast, in the IVM-treated cells, an important accumulation of the GFP signal was observed in the cytoplasm, significantly reducing the Fn/c ratio between the vehicle (42.60±3.61) and IVM-treated cells (1.730±0.17). Indicating that IVM can inhibit the importin *αβ*1 pathway-dependent active nuclear transport of Huh-7 cells.

On the other hand, during DENV-2 infection, IVM treatment did not impair the nucleolar localization of the capsid, showing no difference between the vehicle (6.966±2.02) and IVM treatment (3.645±0.42) (Fig. 4d); the opposite effect was observed with MET treatment (Figs2  3). Specifically, IVM treatment partially impaired the nuclear transport of NS5 (Fig. 4f), as evidenced by a significant reduction in the Fn/c ratio between vehicle-treated (224.6±5.79) and IVM-treated cells (4.160±0.43). Additionally, we increased the IVM concentration to assess whether complete inhibition of NS5 nuclear localization could be achieved. However, we found that at these concentrations, IVM significantly reduced DENV-2 viral litres (Fig. 4g), which is consistent with its well-documented antiviral activity against DENV infection [27]. Taken together, our results suggest that the capsid protein is imported into the nucleus of Huh-7 cells independent of the classical active nuclear transport pathway.

## Discussion

Multiple viral proteins are imported to the nucleus during viral infection, suggesting the role of cellular nuclear transport in viral replication [28,31, 32]. Consequently, several nuclear transport inhibitor drugs with antiviral activity have been proposed, focusing on blocking active nuclear transport [3,27, 33]. However, little is known about passive nuclear transport inhibitors.

It is known that proteins with a molecular weight less than 60 kDa can diffuse into the nucleus through the NPC [34]. In this sense, it was reported that biguanides restrict passive nuclear transport [19]. In addition, previous reports show that MET biguanide at 10 mM reduces DENV replication by activating AMPK and reducing HMGCR activity [21,35]. However, no studies have associated biguanide’s effects on the nuclear transport of DENV proteins. As a first observation of our study, we found that treatment with 1 mM MET induced changes in the nuclear membrane surface of Huh-7 mock-infected and DENV-2-infected cells, missing dark spaces of the pore-like structures (Fig. 1). However, we cannot assert changes directly in the NPCs because the resolution of our microscope was not able to acquire the NPCs.

Furthermore, during DENV-2 infection, we found that MET treatment promoted cytoplasmic retention of the capsid protein in Huh-7 and Vero cells (Fig. 2). Likewise, the same effect was observed during DENV-4 Huh-7-infected cells treated with MET. In contrast, when we replicated this assay in the BHK-21 cell line, MET treatment did not affect the nuclear localization of the capsid protein in both DENV-2 and DENV-4 (Fig. S2), suggesting that nuclear transport of DENV proteins is differential according to cell line type. Likewise, [13] reported that DENV capsid mutated in the putative NLS exhibited different localizations in two cell lines [13]. Not ruling out the passive nuclear transport of capsid since the protein is small enough (14 kDa) to occur [13]. In this sense, Sallaberry *et al*. (2021) demonstrated that the DENV capsid protein shuttles from and to the nucleus by free diffusion [34], supporting our hypothesis that MET inhibits its passive nuclear transport.

As noted in previous studies, ATP modulation can alter passive nuclear transport by reducing both the diameter and height of NPCs [19,30, 36]. This effect has been attributed to the absence of energy-dependent nuclear deformation forces mediated by microtubules [36]. Among the key molecular mechanisms of MET is the reduction of ATP production through interaction with the respiratory chain complex [37,38], implying that MET could affect the passive nuclear transport of capsid, mainly in cells of hepatic origin, as we observed in our results in Huh-7 cells (Fig. 2). Additionally, MET did not affect the nuclear transport of the NS5 protein, suggesting that MET does not affect the active nuclear transport of NS5 since it is well-known that NS5 possesses NLS, which allows its interaction with karyopherins facilitating its nuclear transport [6,31, 39]. In line with the hypothesis that MET could affect passive nuclear transport through its effect on ATP modulation, we inhibited the ATP regenerative system with a non-hydrolyzable ATP analogue known as AMP-PNP. Our results showed that AMP-PNP treatment promoted cytoplasmic retention of capsid in Huh-7 cells (Fig. 3); likewise, AMP-PNP did not affect the nuclear localization of NS5 (Fig. S3), consistent with our MET treatment results.

Similarly, nuclear transport of the 14 kDa HIV-1 Tat protein was reported to be blocked by AMP-PNP, as well as during the absence of ATP, indicating that nuclear transport of small proteins could be ATP-dependent [40], hinting at the inhibition of passive nuclear transport. However, the results of AMP-PNP treatment obtained in this work should be taken cautiously, given that the effect of the non-hydrolyzable ATP analogue inhibits the total ATP regenerative system of the cell and not only aspects related to nuclear transport. Furthermore, it is noteworthy that AMP-PNP treatment harmed DENV-2 infection, resulting in a decrease in viral proteins and viral litres (Fig. 3). On the other hand, treatment with 1 mM MET did not significantly affect DENV-2 replication but modified nuclear capsid transport; in contrast, higher concentrations of MET inhibit viral replication [20]. Although MET blocks cholesterol synthesis by activating AMPK and reducing HMGCR activity [41], our data provide evidence that this hypoglycaemic drug also impairs the nuclear transport of viral proteins, particularly the capsid protein. On the other hand, cholesterol inhibition by lipid-lowering drugs such as ATV affects DENV replication [42], and it also impairs the nuclear transport of DENV-2 viral protease and viral polymerase [2] because ATV decreases the prenylation of small GTPases involved in intracellular transport [43]. Increasingly, we are uncovering the pleiotropic effects of FDA-approved drugs, highlighting that nuclear transport of viral proteins is necessary in the replicative cycle.

**Fig. 5.**
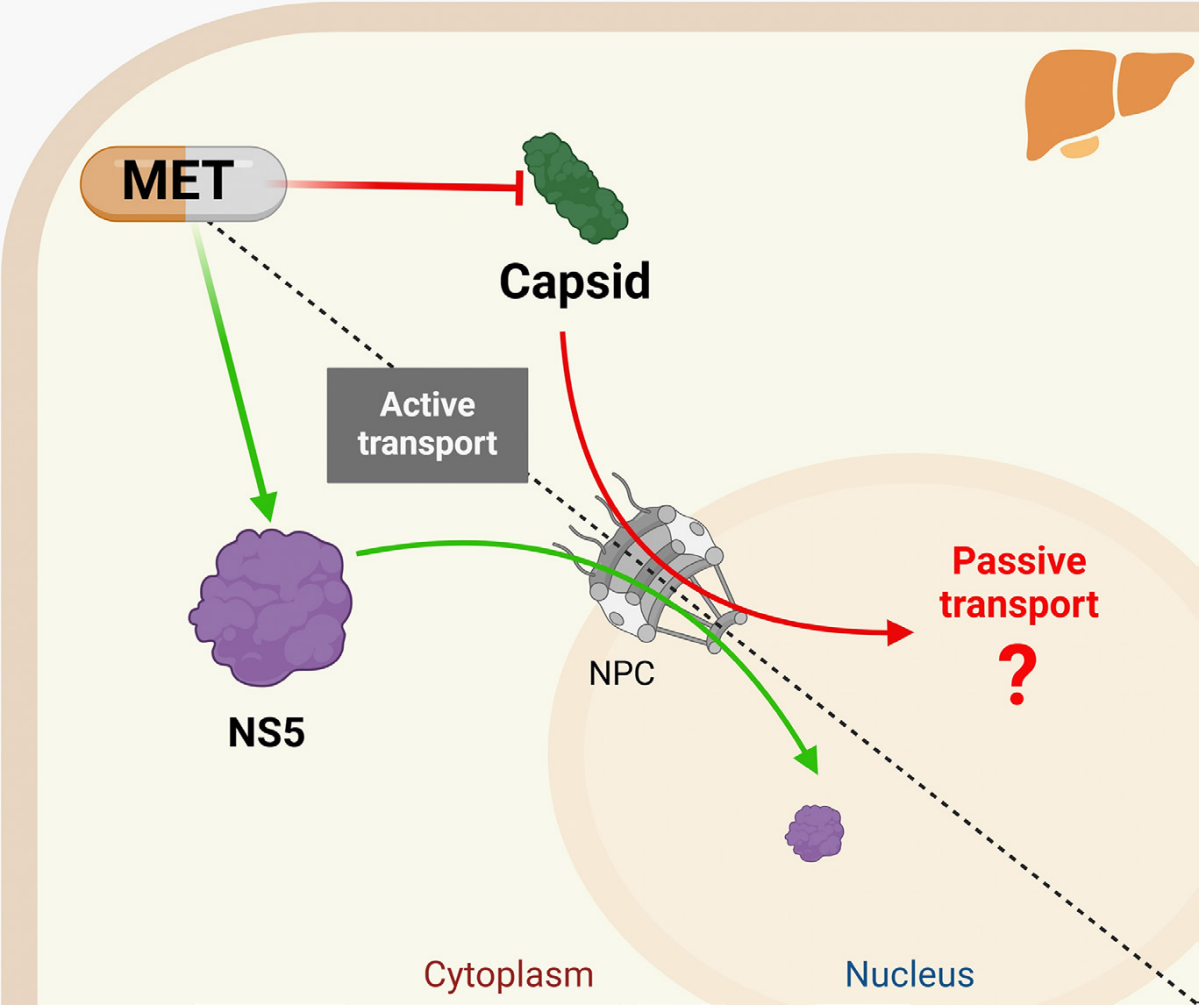
Proposed scheme of the MET effect on capsid and NS5 nuclear transport. MET treatment impairs the nuclear import of DENV-2 capsid in Huh-7 cells without affecting NS5 active nuclear transport.

Bhuvanakantham *et al*. proved that the West Nile virus capsid protein is transported to the nucleus by directly interacting with importin-*α* [44]. By mutagenesis studies, it has been shown that DENV capsid possesses functional NLS dependent on the classical importin *αβ*1 pathway [13]. Nonetheless, our results of nuclear transport inhibition dependent on the importin *αβ*1 pathway using IVM partially affected the nuclear transport of the NS5 protein but did not impair capsid nuclear transport in Huh-7 cells, showing its typical nucleolar localization (Fig. 4), indicating that capsid protein transport is not via importin *αβ*1 in our cell hepatic line, pointing out that its nuclear transport depends on non-classical pathways, without ruling out its passive nuclear transport. Finally, these data support the idea that MET treatment could retain the DENV-2 capsid protein in the cytoplasm by the cell bioenergetics MET effect, which promotes changes in the NPC, reducing passive nuclear transport, as shown in our proposed model (Fig. 5).

## supplementary material

10.1099/jgv.0.002089 Uncited Supplementary Material 1.

## Abbreviations

AMPK: AMP-activated protein kinase
ATV: atorvastatin
BCA: bicinchoninic acid
BHK-21: baby hamster kidney-21 cells
CMC: carboxymethylcellulose
DENV: dengue virus
DMEM: Dulbecco’s Modified Eagle Medium
FDA: Food and Drug Administration
Fn/c: fluorescence nucleus–cytoplasmic
GFP: green fluorescent proteins
GTP: Guanosine Triphosphate
HMGCR: HMG-CoA reductase
IVM: ivermectin
MET: metformin
MFI: mean fluorescence intensity
MTT: 3-(4,5-Dimethylthiazol-2-yl)-2,5-diphenyltetrazolium bromide
NLS: nuclear localization sequence
NPCs: nuclear pore complexes
PFU: plaque-forming units
SEM: scanning electron microscopy
SV40: simian virus 40
