# Supplementary material for: Cytoplasmic retention of dengue virus capsid protein by metformin impairing nuclear transport
Source: J Gen Virol. 2025 Mar 20;106(3):002089. doi: 10.1099/jgv.0.002089 (PMC11926096; doi:10.1099/jgv.0.002089)
Supplement: Uncited Supplementary Material 1. [file jgv-106-02089-s001.pdf]

Table S1. Primary and secondary antibodies.

Antibody	WB dilution	IF dilution	Catalog and source
Mouse anti-CRT*	1:1000	-	Santa Cruz, Cat# sc373863
Mouse anti-LMNA*	1:1000	-	Santa Cruz, Cat# sc376248
Mouse anti-GAPDH*	1:1000	-	Santa Cruz, Cat# sc47724
HRP-conjugated goat anti-mouse**	1:5000	-	Cell Signaling, Cat# 7076S
HRP-conjugated goat anti-rabbit**	1:5000	-	Cell Signaling, Cat# 7074S
Rabbit anti-C*	1:5000	1:300	GENETEX, Cat# GTX103343
Rabbit anti-NS5*	1:5000	1:300	GENETEX, Cat# GTX124253
Goat anti-mouse Alexa Fluor488***	-	1:750	Invitrogen, Cat# A21202
Goat anti-rabbit Alexa Fluor 555***	-	1:300	Invitrogen, Cat# A21428

* Primary antibodies were incubated overnight (16-18 hours) at 4°C.

** Secondary antibodies for Western blotting were incubated at room temperature for 1 h.

*** Secondary antibodies for indirect immunofluorescence were incubated at 37°C for 2 h.

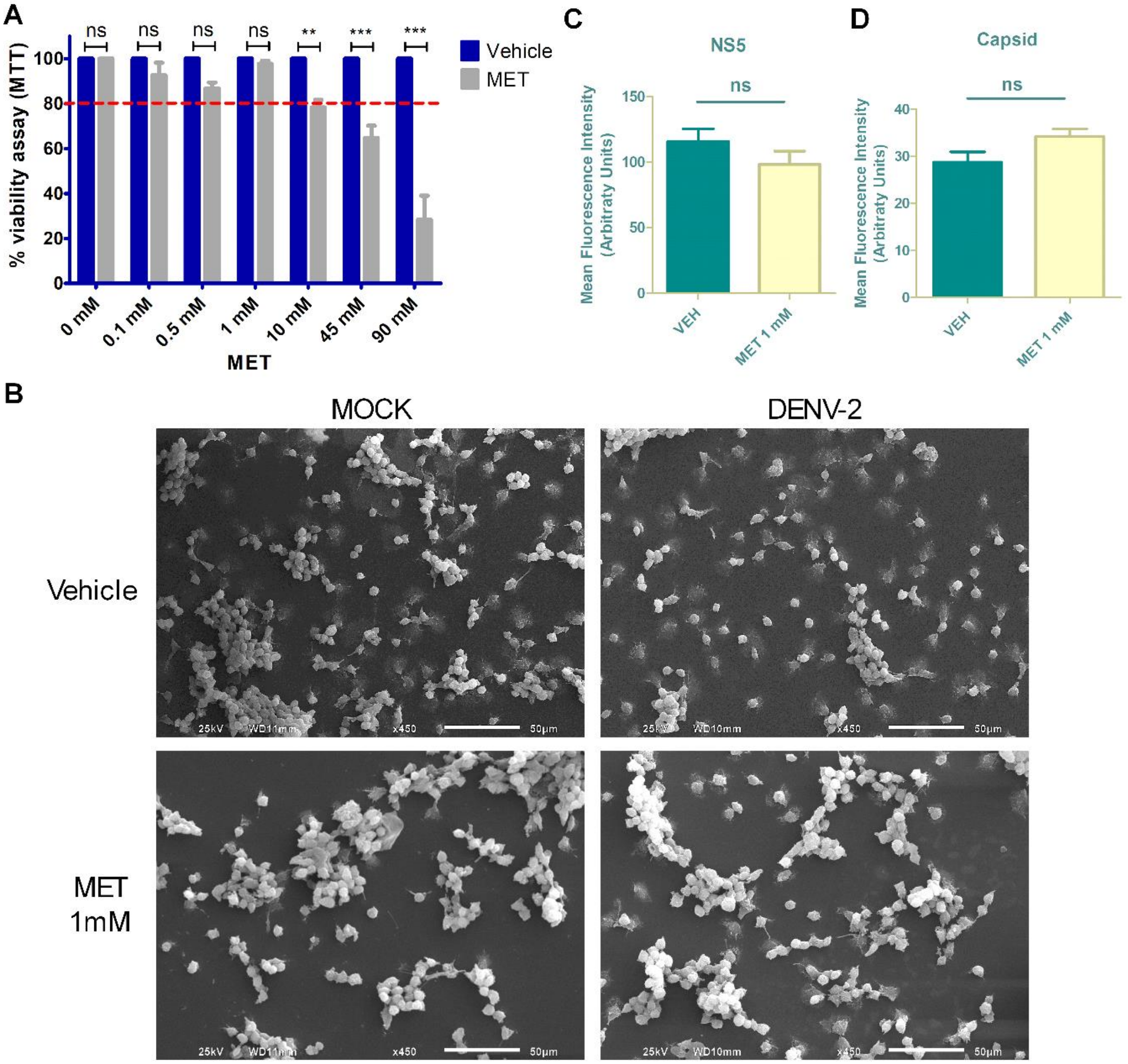

Figure S1. Cell viability assay and representative fields of Huh-7 cell nuclei viewed by SEM.

(A) Percentage of cell viability of MET treatment at 24 hours from three independent experiments in triplicate. Data were represented as mean ± SEM. Two-way ANOVA performed a statistical comparison with Sidak's multiple comparison test. ns, not significant; $**p < 0.01$, $***p < 0.001$. (B) Magnification x450 of nuclei isolated from uninfected and DENV-2-infected Huh-7 cells treated with 1mM MET. Scale bar, 50 µm. (C and D) The total MFI of NS5 and capsid were analyzed. Data correspond to the mean of three independent experiments, and significant differences were obtained from the students' t-tests. Error bars represent the SEM. ns, not significant.

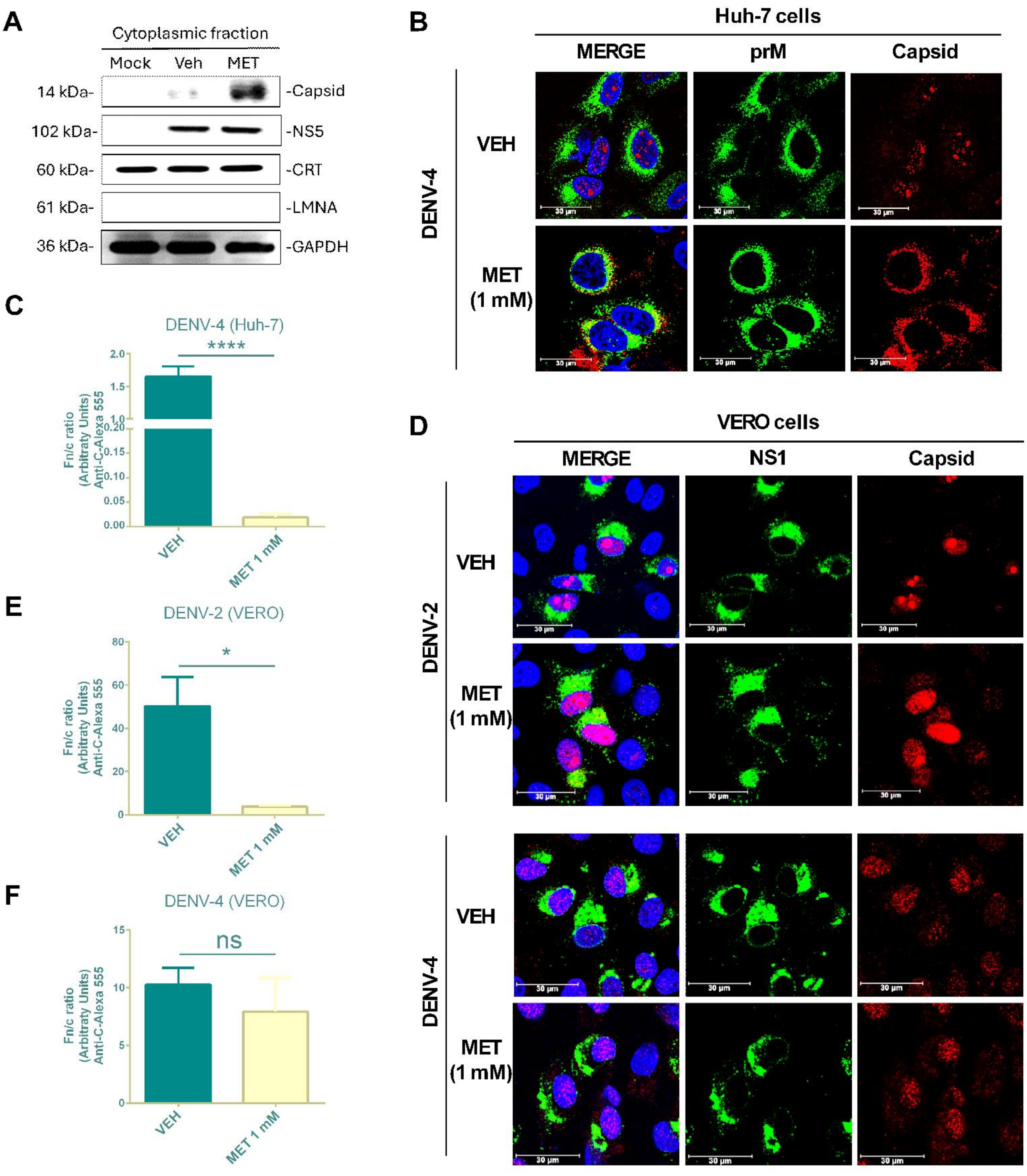

Figure S2. Subcellular localization of DENV-2 and DENV-4 capsid in Vero cell line during MET treatment.

(A) Western blot analysis of the cytoplasmic fraction of Huh-7 cells infected with DENV-2 and treated or not with 1 mM MET for 24 h. CRT and LMNA were used as purity controls, and GAPDH was used as a loading control. (B) Cytoplasmic and nuclear localization of capsid protein by confocal microscopy images of DENV-4-infected Huh-7 cells treated with 1mM MET. (C) MFI graphic showing the Fn/c ratio between vehicle-treated and 1 mM MET-treated DENV-4 Huh7 infected cells. (D) Cytoplasmic and nuclear localization of capsid protein by confocal microscopy images of DENV-2 or DENV-4-infected Vero cells treated with 1mM MET. (E and F) MFI graphic showing the Fn/c ratio between vehicle-treated and 1 mM MET-treated DENV-2 or DENV-4 Vero infected cells. Data correspond to the mean of three independent experiments, and significant differences were obtained from the students' t-tests. Error bars represent the SEM. ns, not significant; * p 0.05; **** p< 0.0001. Scale bar, 30 µm.

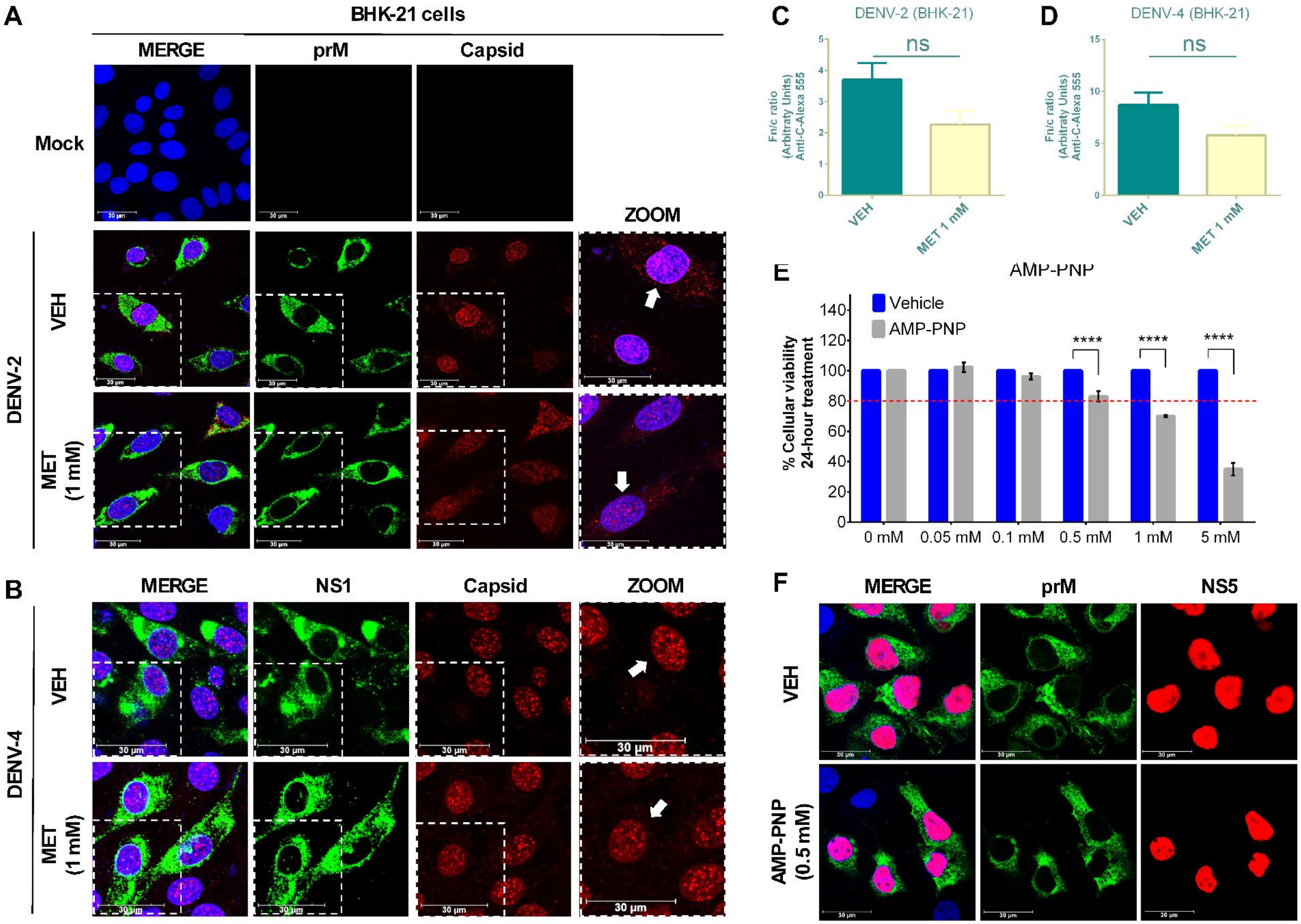

A
BHK-21 cells
MERGE
prM
Capsid
Mock
ZOOM
DENV-2
VEH
MET (1 mM)
30 µm
B
MERGE
NS1
Capsid
ZOOM
DENV-4
VEH
MET (1 mM)
30 µm
C
DENV-2 (BHK-21)
ns
Fn/c ratio (Arbitraty Units) Anti-C-Alexa 555
VEH
MET 1 mM
D
DENV-4 (BHK-21)
ns
E
AMP-PNP
Vehicle
AMP-PNP
% Cellular viability 24-hour treatment
****
0 mM
0.05 mM
0.1 mM
0.5 mM
1 mM
5 mM
F
MERGE
prM
NS5
VEH
AMP-PNP (0.5 mM)

Figure S3. Subcellular localization of DENV-2 and DENV-4 capsid in BHK-21 cell line during MET treatment and effect of AMP-PNP on cell viability and subcellular localization of NS5 protein.

(A and B) Cytoplasmic and nuclear localization of capsid protein by confocal microscopy images of DENV-2 or DENV-4-infected BHK-21 cells treated with 1mM MET. (C and D) MFI graphic showing the Fn/c ratio between vehicle-treated and 1 mM MET-treated DENV-2 or DENV-4 BHK-21 infected cells. Data correspond to the mean of three independent experiments, and significant differences were obtained from the students' t-tests. (E) Percentage of cell viability of AMP-PNP treatment at 24 hours from three independent experiments in triplicate. Data were represented as mean ± SEM. Two-way ANOVA performed a statistical comparison with Sidak's multiple comparison test. ns, not significant; $****p < 0.0001$. (F) Nuclear localization of NS5 protein by confocal microscopy images of DENV-2-infected Huh-7 cells treated with 0.5 mM AMP-PNP.
